# Teachers’ Judgments of Students’ School-Wellbeing, Social Inclusion, and Academic Self-Concept: A Multi-Trait-Multimethod Analysis Using the Perception of Inclusion Questionnaire

**DOI:** 10.3389/fpsyg.2020.01498

**Published:** 2020-07-07

**Authors:** Susanne Schwab, Ghaleb Hamad Alnahdi

**Affiliations:** ^1^Centre for Teacher Education, University of Vienna, Vienna, Austria; ^2^Research Focus Area Optentia, North-West University, Vanderbijlpark, South Africa; ^3^Department of Special Education, College of Education, Prince Sattam Bin Abdulaziz University, Al-Kharj, Saudi Arabia

**Keywords:** school well-being, social inclusion, academic self-concept, perceptions of inclusion, teachers’ judgments, MTMM

## Abstract

Students’ school well-being, social inclusion, and academic self-concept are considered important outcome variables of schools. In the present study, these three variables were examined from teachers’ and students’ perspective (grades 5–9). The aim of the study was to investigate the construct validity (convergent and discriminant validity) of the teacher’s version of the Perception of Inclusion Questionnaire (PIQ). Further, we investigated whether or not it is meaningful to include the perspective of a second teacher. The dataset consists of PIQ ratings of 151 students as well as ratings from two main subject teachers. The results for psychometric properties show that the students’ as well as the teachers’ version of the PIQ is suitable for secondary school students. The confirmatory factor analyses demonstrated good model fit for the three-dimensional factorial structure. By excluding one teacher’s rating from the model, the multitrait-multimethod analysis provided indicators for the PIQ’s construct validity (convergent and discriminant validity) of the traits and discriminant validity of the methods.

## Introduction

Educating all students together in the same class has become a shared goal in Europe (for an overview, see e.g., Schwab, 2020). This is evident in European politics (Watkins, 2017) and the decreasing number of students in exclusive school settings (European Agency for Special Needs and Inclusive Education, 2018). This major change in the education system needs to be evaluated to determine the advantages of inclusive education and identify challenges. Inclusive education is not simply the education of all students most of the time in the same classroom. It also refers to equitable quality education and the avoidance of learning barriers (United Nations Educational Scientific and Cultural Organization, 2017). Currently, research in the field of inclusive education focusing on diverse outcomes (e.g., academic outcome, social-emotional outcome) is limited. When addressing research about inclusive education, not only students’ academic achievement but also their feelings need to be taken into account. Several researchers claim (e.g., Bourke and Mentis, 2013; De Leeuw et al., 2018) the importance of listening to students’ voices and including them into research, especially in the field of inclusion. “We need to spend more time listening to and trying to understand the perspectives of self-advocates” (Giangreco et al., 2001, p. 59).

### Students’ Perception of Inclusive Education

The three variables, which are related to students’ feelings about inclusive education are: social inclusion, emotional well-being, and academic self-concept. Previous reviews indicate that students with SEN feel a lower level of social inclusion than their peers without SEN (Koster et al., 2009; Bossaert et al., 2013; Schwab, 2018). However, the effect size of group differences between students with and without SEN to a large extent depends on the instrument—according to students’ self-rated social inclusion. Sometimes, no differences can be identified. When considering the emotional well-being of students with and without SEN, some study outcomes did not discover lower school well-being for students with SEN educated in mainstream classes than for their peers without SEN (Schwab et al., 2015), although others did (McCoy and Banks, 2012; Skrzypiec et al., 2016). Studies about the academic self-concept demonstrated a lower academic self-concept of students with SEN in inclusive classes compared to their peers without SEN (e.g., Bear et al., 2002; Weber and Freund, 2017).

### Teachers’ Judgments

Especially in the school context, it can be meaningful to include multi-informant assessments as on the one hand students might behave differently in different situations (e.g., when the teacher is on-site) and because on the other hand raters might perceive the same behavior differently (see, e.g., Achenbach, 2018). As van der Ende et al. (2012), p. 293) stated: “Each informant contributes unique information about an individual’s problems.” Moreover, as students’ social inclusion, emotional well-being and academic self-concept are important outcome variables of inclusive education, teachers’ needs to be aware of students’ feelings toward them. The more accurate judgments teachers can make about students’ feelings, the better they support the students’ development. For instance, teacher behavior such as the feedback a teacher gives can influence students’ social participation (Huber et al., 2018). Further, teachers’ feedback is related to students’ intension to quit school (Schwab et al., 2019) as well as students’ academic self-concepts (Schwab et al., 2019). Considering interrater agreement between students’ and teachers’ ratings of non-academic variables, previous studies showed a rather low overlap. For instance, Urhahne and Zhu (2015b) found that the overlap between students’ and teachers’ ratings of academic self-concept ranged between 0.30 and 0.60. For students’ well-being, Urhahne and Zhu (2015a) found a low to moderate overlap between students’ and teachers’ ratings. Similarly, relatively low accuracy for teacher ratings has been found for students’ social inclusion (e.g., Südkamp et al., 2018).

While plenty of research has been conducted on the accuracy of teacher ratings with students’ outcomes or ratings, we do not know much about the ratings of two different teachers. Schwab and Gebhardt (2016) investigated the judgments of regular and special needs teachers of students’ social participation. The correlation of the overall score of social inclusion between the two teachers’ ratings was *r* = 0.43. They further interpreted social inclusion at three levels (lowest level indicated that the student is an outsider, middle level that the student is accepted, and the highest level that the student has friends in class). In more than 70% of the cases, both teachers rated the social participation at the highest level. However, in around 16% of the cases, they differed in one level, and in around 13% of the cases, the two teachers had opposite opinions: one rated students’ social inclusion at the highest level while the other indicated the lowest. The results of Schwab and Gebhardt (2016) indicated that regular and special needs teachers might have different insights in students’ social inclusion. However, it might also be the case that these two teachers do not spend the same amount of time with the students. It can be assumed that regular teachers spent more time with students without SEN and special need teachers spend more time with the students with SEN. In some cases, they might even spend part of the school time outside in a different resource room. Therefore, it would be interesting to check if the overlap of teachers’ ratings between two regular teachers would be higher.

### The Perception of Inclusion Questionnaire (PIQ)

To assess students’ social inclusion, school well-being, and academic self-concept from students’ and teachers’ perspective, appropriate screening instruments are required. These instruments must be free of charge, easy to use, and processing must not take much time. One instrument that adheres to these criteria is the Perception of Inclusion Questionnaire (PIQ; Venetz et al., 2015). The PIQ is a short screening instrument available in student, teacher, and parents/caregiver versions in more than 20 languages (e.g., English, German, French, and Spanish). It considers all three subthemes (social inclusion, school well-being, and academic self-concept), takes around 5 min to complete, and is accessible online.^1^ Previous studies already demonstrated that it can be used for different subsamples (e.g., students with and without SEN; Zurbriggen et al., 2017; DeVries et al., 2018; Knickenberg et al., 2019). DeVries et al. (2018) concluded from the results of their psychometric analysis that the PIQ meets high qualities and therefore is suitable for use in the context of inclusive education. However, these studies only focused on the students’ version of the PIQ. For primary students, Schwab et al. (submitted) showed that the teacher version of the PIQ fits with high psychometric properties and that it is meaningful to include teachers’ ratings as the overlap between students’ and teacher ratings was not very high. Within this article, also mothers’ and fathers’ ratings of the PIQ were analyzed. One study by Venetz et al. (2019) also verified the psychometric quality of the teachers’ version for secondary school students, confirming the three-dimensional factor structure and high reliability thereof. Moreover, they checked for the overlap between students’ ratings and teachers’ judgments. The results of a correlated trait-correlated method minus one model indicated a low overlap between students’ and teachers’ ratings. The method-specificity for teacher reports was high, while the consistency was low. However, to date, no study is available, which confirmed the results of Venetz et al. (2019), and in addition, no study included the perspective of a second teacher.

### Present Study

This study aims to replicate the results of Venetz et al. (2019) by investigating the psychometric qualities of the teachers’ version of the PIQ for secondary school students. As the results about the psychometric qualities of the teachers’ version (for secondary school students) of the PIQ are limited to the study of Venetz et al. (2019), we will test the reliability as well as the factor structure of this instrument. Moreover, leaning on the work of Venetz et al. (2019), the overlap between students and teachers will be examined. In addition, it will be analyzed if it is meaningful to include the perspective of a second teacher. Therefore, the previous work of Schwab and Gebhardt (2016) will be extended for school well-being and students’ academic self-concepts—as the study of Schwab and Gebhardt (2016) only focused on the overlaps of students’ and teachers’ perceptions of students’ social inclusion.

## Materials and Methods

### Participants

Data from this study were collected from a larger sample. In total, 18 schools (*N* = 42 school classes) in North Rhine-Westphalia, a federal state in Germany, participated in the paper–pencil survey. Only classes in which at least one student had been officially diagnosed as having special educational needs were invited to participate in the study. The schools prepared for different pathways from higher education to vocational education, were located in urban and rural areas, and varied in terms of socio-economic status. Students attended grades 5–9 and were aged between 10 and 17 years. In addition to the students, two main subject teachers (German, English, or Mathematics subject teachers) per class were invited to fill out the questionnaires for the students.

However, for the present study, only a subsample of ten classes was used as only for ten out of the 42 classes PIQ reports from students and two main subject teachers were available (as in some classes, teachers were not able to fill out PIQ reports for all of their students). Therefore, the current study included data from 151 students (86 male and 65 female students). Moreover, around 30% of these students did not speak German as their first language. Around 14% of the students were officially diagnosed having special educational needs. The majority of students (61.6%) attended a *Realschule* or *Hauptschule* (20.5%), which prepares for vocational training; 13.9% a *Gymnasium*, which prepares students for university; and 4% a *Gesamtschule*, which prepares them for vocational training and university.

### Instrument

In addition to socio-demographic questions (e.g., gender, having SEN), students filled out the German language self-report scale of the PIQ (Venetz et al., 2015) (see text footnote 1). The instrument assesses students’ perception of their emotional, social, and competence-based relatedness and can be used for students from grades 3–9. All dimensions are measured via four items rated on a four-point Likert scale (e.g., “I like going to school” for school well-being, “I have very good relationships with my classmates” for social inclusion, and “I do well in my schoolwork” for academic self-concept). High psychometric properties for the student version have been confirmed several times (e.g., Zurbriggen et al., 2017; DeVries et al., 2018; Venetz et al., 2019). The teachers’ version of the PIQ (Venetz et al., 2015) was used for teachers’ ratings of students’ social inclusion, school well-being, and their academic self-concept. As mentioned above, the psychometric properties for the teachers’ version have only been confirmed in one study by Venetz et al. (2019) for secondary school teachers and in one study by Schwab et al. (submitted) for primary school teachers.

### Analyses

Different statistical analyses were employed in this study. A reliability analysis, correlations, and confirmatory factor analyses (CFA) were performed to check whether the three-dimensional factor structure could be confirmed. Following the guidelines of Hu and Bentler (1999), model fit was estimated using a chi-square test, comparative fit index (CFI > 0.95), and root mean square error of approximation (RMSEA < 0.06). Moreover, a multitrait-multimethod (MTMM) analysis was conducted to estimate the convergent and discriminant validity of the two versions of PIQ (self-reports and teachers’ ratings). The MTMM analysis is one of the methods most used to examine the validity of psychological measures (Hintz et al., 2019). Three methods were used to collect data (students’ self-reports, ratings of teacher 1, and ratings of teacher 2), and three traits were measured (traits refer to the three subscales of the PIQ: social inclusion, school well-being and academic self-concept.) This satisfies the requirement for an MTMM analysis to have at least two methods and two traits (Campbell and Fiske, 1959). The MTMM analysis began by using the MTMM matrix developed by Campbell and Fiske (1959), a practical method to examine the construct validity of measures in terms of convergent and discriminant validity. One advantage of an MTMM analysis is that it enables researchers to examine latent traits factors from different sources (wanted) and whether a latent method factor (unwanted) exists (Koch et al., 2015). Widaman (1985) proposed examining convergent and discriminant validity through significance tests of the differences between nested models, which we followed as a guideline in this study. This lead to the popular CFA approach to MTMM analyses (CFA-MTMM) (Byrne, 2010). In this study, the terms MTMM and CFA-MTMM are used interchangeably to refer to the CFA approach to MTMM analyses.

In total, four models were computed for the CFA-MTMM analyses. The first model (M1) has no constrains on traits or methods. The second model (M2) includes only methods and no traits. In Model 3 (M3), the correlation between traits was set as perfect (1) using the freely correlated method. In the last model (M4), traits were freely correlated, while the correlation among methods was constrained as zero (no correlation). The application of the CFA to the MTMM matrices is a sophisticated method for evaluating the construct validity of scales through estimating convergent validity, discriminant validity, and the method effect (Brown, 2014). Following the guidelines of Widaman (1985) is the common practice to achieve this goal (Byrne, 2010). “As such, the hypothesized MTMM model is compared with a nested series of more restrictive models in which specific parameters either are eliminated or are constrained equal to zero or 1.0. The difference in χ2 (Δχ2) provides the yardstick by which to judge evidence of convergent and discriminant validity” (Byrne, 2010, p. 276). All analyses were conducted with Amos 20 with a maximum likelihood estimation.

## Results

### Descriptive Statistics, Reliability, and Intraclass Correlations

Table 1 presents the mean and standard deviations of students’ and teachers’ ratings for the three subscales of the PIQ. In general, all means are high, as the theoretical mean of the scale was 2.5. The internal consistencies of the student sample were acceptable for the subscales school well-being and social inclusion, but low for academic self-concept. For the ratings of both teachers, the reliability values were high (0.84 ≤ α ≤ 0.90). Furthermore, intraclass correlations (ICC) were calculated for the ratings of the two teachers. The correlations were 0.87 for school well-being, 0.85 for social inclusion, and 0.90 for academic self-concept.

**TABLE 1:** Participants’ mean and standard deviation scores, reliability of PIQ scales (Cronbach’s alpha coefficient), and intraclass correlation (ICC).

**Subscale**	***N***	***M***	**SD**	**Cronbach’s alpha**	**ICC**
**Student**					
School Well-Being (SWB)	149	3.00	0.70	0.81	
Social Inclusion (SI)	150	3.32	0.58	0.74	
Academic Self-Concept (ASC) (AC)	149	2.88	0.52	0.61	
**Teacher 1**					
School Well-Being (SWB)	151	3.02	0.54	0.90	
Social Inclusion (SI)	151	3.07	0.58	0.85	
Academic Self-Concept (ASC)	151	2.59	0.73	0.87	
**Teacher 2**					
School Well-Being (SWB)	151	3.10	0.49	0.90	
Social Inclusion (SI)	151	3.04	0.54	0.84	
Academic Self-Concept (ASC)	151	2.65	0.65	0.88	
**Teacher 1/Teacher 2**					
School Well-Being (SWB)					0.874
Social Inclusion (SI)					0.847
Academic Self-Concept (ASC)					0.897

#### Factor Analyses

First, CFA analyses were conducted for all three samples separately as a prerequisite step to any further analysis. Table 2 shows the model fit indices for all samples. For the students’ data, the first model showed acceptable fit indices, such as the comparative fit index (CFI) = 0.94, Tucker–Lewis index (TLI) = 0.92, and root mean square error of approximation (RMSEA) = 0.06. For the second dataset for teacher 1, also acceptable fit indices were obtained: CFI = 0.96, TLI = 0.94, and RMSEA = 0.08. For the teacher 2 dataset, the fit indices were acceptable too: CFI = 0.97, TLI = 0.96, and RMSEA = 0.06. In sum, the values were in the acceptable range, indicating that the data for all three samples fit the three-factor structure of the PIQ.

**TABLE 2:** Fit indices for PIQ for all three samples (student sample, teacher 1, and teacher 2).

**Model**	**χ^2^**	***df***	***p***	**CFI**	**GFI**	**TLI**	**RMSEA**	**90% CI**
CFA (students)	80.942	50	0.004	0.937	0.915	0.917	0.064	[0.037,0.089]
CFA (teacher 1)	98.402	49	0.000	0.956	0.900	0.940	0.082	[0.058,0.105]
CFA (teacher 2)	77.242	49	0.006	0.974	0.918	0.965	0.062	[0.032,0.087]

*MTMM, multitrait-multimethod; χ^2^, chi-square statistic; CFI, comparative fit index; GFI, goodness of fit index; TLI, Tucker–Lewis index RMSEA, root mean square error of approximation; CI, confidence interval.*

### Correlations

Before performing the CFA-MTMM, the correlation matrix for traits was checked. According to Campbell and Fiske (1959), the correlations between the same traits and different methods should be strong. Table 3 shows that all nine correlations were statistically significant, thus supporting the convergent validity of the traits (all correlations are significant at *p* < 0.01).

**TABLE 3:** Correlations of students’ and teachers’ reports (trait correlations).

	**Correlation of students’ and teachers’ reports (teacher 1)**	**Correlation of students’ and teachers’ reports (teacher 2)**	**Correlation of teachers’ reports (teacher 1 x teacher 2)**
School Well-Being (SWB)	0.325**	0.255**	0.377**
Social Inclusion (SI)	0.371**	0.386**	0.412**
Academic Self-Concept (ASC)	0.333**	0.348**	0.583**

**Correlation is significant at the 0.01 level (2-tailed).

As an indicator of discriminant validity, the correlations of different traits based on different methods should be lower than those for the same traits based on different methods. In the case of no method variance, different traits based on the same method should be similarly correlated to different traits using different methods. However, the correlations between different traits from the same method were higher than that between different traits from different methods. In addition, in the ideal construct validity situation (good convergent and discriminant validity), it is assumed that all items correlate more highly with the traits than with the methods. However, 20 out of the 36 items (12 items from each method) correlate highly with the methods, rather than the traits.

### Multitrait-Multimethod Models

**FIGURE 1:**
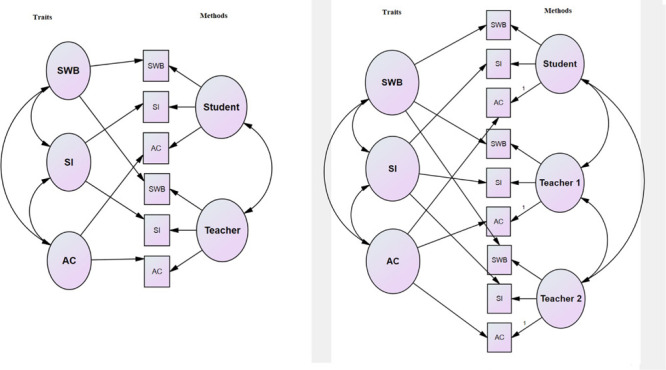
Model with three methods (student, teacher 1, and teacher 2) (right) and model with two methods (student and teacher) (left). SWB, school well-being; SI, social inclusion; ASC, academic self-concept.

A CFA with a MTMM model was performed to examine the construct validity (convergent and discriminant validity) of two versions of the PIQ (see Figure 1).

Four models were computed for the CFA-MTMM analysis. The first model (M1) had no constrains on traits or methods. The second model (M2) included only methods and no traits. In Model 3 (M3), the correlation between traits was set as perfect (1) with a freely correlated method. In the last model (M4), traits were freely correlated, while the correlation for methods was constrained as zero (no correlation). Table 4 indicates that Model 1 (M1) has the best fit indices compared to the other three models. This shows that two of the three indicators needed to confirm convergent and discriminant validity were established. The only indicator that did not support validity was when M1 demonstrated better fit than M4, and thus did not have equal fit.

**TABLE 4:** Goodness-of-fit indices for MTMM models.

**Model**		**χ^2^**	***df***	**CFI**	**RMSEA**	**90% CI**
M1	Freely correlated traits; freely correlated methods	838.152	549	0.904	0.059	[0.051,0.067]
M2	No traits; freely correlated methods	1934.054	591	0.556	0.123	[0.117,0.129]
M3	Perfectly correlated traits; freely correlated methods	1162.674	552	0.798	0.086	[0.079,0.093]
M4	Freely correlated traits; uncorrelated methods	878.090	552	0.892	0.063	[0.055,0.070]

*MTMM, multitrait-multimethod; χ^2^, chi-square statistic; CFI, comparative fit index; RMSEA, root mean square error of approximation; CI, confidence interval.*

Differences were evident in the chi-square (Δχ^2^) test performed to compare the data fit between the two models. A significant Δχ^2^ means that the first model is better than the other one. In addition, another indicator is the difference in the CFI (Δ CFI) of M1 and other models (Cheung and Rensvold, 2002; Dimitrov, 2010). Here, a Δ CFI ≥ 0.01 indicates significant differences in fit between the two models. Table 5 shows a significant Δχ^2^ between the two models (M1 and M2) and a large difference in CFI = 0.348. Both support the convergent validity of the subscales (Byrne, 2010). In other words, the model that includes traits significantly explains the data better than the model without them (see Table 5).

**TABLE 5:** Comparison of goodness-of-fit indices.

	**Three methods**		
**Model comparisons**	**Δχ2**	**Δ df**	**Δ CFI**
Test of convergent validity	1095.9**		
Model 1 vs. Model 2 (traits)		42	0.348
Tests of discriminant validity	324.522**		
Model 1 vs. Model 3 (traits)		3	0.106
Model 1 vs. Model 4 (methods)	39.93**	1	0.012

*MTMM, multitrait-multimethod; Δχ^2^, differences in chi-square statistic; Δ CFI, differences in comparative fit index. ^∗∗^*p* < 0.001.*

For the discriminant validity of the traits, M1 demonstrated significantly better fit indices than M3. This supports claims that traits are indicators of discriminant validity.

Next, the fit indices for M1 and M4 were compared. In the comparison, a large χ^2^ difference or CFI difference is evidence of a lack of discriminant validity of the methods. Table 6 indicates that the difference in the results of the chi-square test χ^2^ for M1 and M4 was significant and the change in CFI was more than 0.01 (Δ CFI = 0.012). This implies a greater overlap between the methods than should be. To examine that, we excluded one of the methods (teacher 1 once and teacher 2 in the second analysis) to understand if the discriminant validity of the methods would improve with two methods only. Thus, students and one teacher were included in each follow-up MTMM analysis (see Table 6).

**TABLE 6:** Goodness-of-fit indices for MTMM models (one teacher only each time).

**Model**		**S-B χ^2^**	***df***	**CFI**	**RMSEA**	**90% CI**
M1.1	Freely correlated traits; freely correlated methods (T1)	266.129	222	0.974	0.036	[0.015,0.052]
M1.2	Freely correlated traits; freely correlated methods (T2)	285.005	221	0.962	0.044	[0.027,0.058]
M2.1	No traits; freely correlated methods (T1)	761.263	249	0.695	0.117	[0.108,0.127]
M2.2	No traits; freely correlated methods (T2)	934.477	249	0.582	0.136	[0.127,0.146]
M3.1	Perfectly correlated traits; freely correlated methods (T1)	509.229	225	0.831	0.090	[0.081,0.102]
M3.2	Perfectly correlated traits; freely correlated methods (T2)	515.272	224	0.825	0.093	[0.083,0.104]
M4.1	Freely correlated traits; uncorrelated methods (T1)	278.315	223	0.967	0.041	[0.022,0.055]
M4.2	Freely correlated traits; uncorrelated methods (T2)	298.889	222	0.954	0.048	[0.033,0.062]

*MTMM, multitrait-multimethod; χ^2^, chi-square statistic; CFI, comparative fit index; RMSEA, root mean square error of approximation; CI, confidence interval; T1, teacher 1; T2, teacher 2.*

A significance test for differences in χ^2^ and CFI indicated a similar result to those of the three methods model in terms of indicators of the convergent validity of traits. In addition, similar to the three methods model, the discriminant validity for traits was confirmed by significant χ^2^ differences and differences in CFI larger than 0.01. The discriminant validity for methods improved in the two methods model, with differences in CFI of less than 0.01 between Model 4.1 and Model 1.1, and similar results for Model 4.2 compared with Model 1.2 (see Table 7). This could mean that discrete data can be obtained from these two methods (students and teacher), and each method yields partially different information that can enhance our understanding.

**TABLE 7:** Goodness-of-fit indices comparisons with two methods only (student and only one teacher).

	**Two methods (T1)**			**Two methods (T2)**		
**Model comparisons**	**Δχ2**	**Δ df**	**Δ CFI**	**Δχ2**	**Δ df**	**Δ CFI**
Test of convergent validity	495.134**					
Model 1 vs. Model 2 (traits)		27	0.279*	649.472**	28	0.38*
Tests of discriminant validity	243.1**					
Model 1 vs. Model 3 (traits)		3	0.143*	230.267**	3	0.137*
Model 1 vs. Model 4 (methods)	12.186**	1	0.007	13.884**	1	0.008

MTMM, multitrait-multimethod; Δχ^2^, differences in chi-square statistic; Δ CFI, differences in comparative fit index. **p < 0.001, T1, teacher 1, T2, teacher 2.

## Discussion

The primary objective of this research was to examine the psychometric properties (reliability and factor structure) of the teacher version of the PIQ. As a second objective, the ratings about students’ social inclusion, school well-being, and academic self-concept of two teachers were analyzed.

Consistent with the results of Venetz et al. (2019), the teachers’ version of the PIQ demonstrated high psychometric qualities according to its reliability. However, for all three subscales, the reliability of students’ reports was lower than the reliabilities of both teacher samples. One possible explanation for the lower reliability in the student sample might be that students struggled more with negatively formulated items. Reverse-worded items can cause problems for student samples, as they might struggle to understand them. According to the factor structure, the expected three-dimensional factor structure was confirmed for the all three samples, the students’ self-reports, the ratings from teacher 1 as well as the ratings from teacher 2. As a side note, the descriptive results confirm that in general most students have high levels of school well-being, social inclusion, and academic self-concept. Therefore, the instrument should be used as a screening tool. In practice, teachers need to closely examine students with a low score.

The overlap between students’ and teachers’ ratings clearly shows that using teacher’s judgment as a surrogate for student’s perception is not a proper measure. However, utilizing data from different sources helps to increase our understanding of the variables we study. Especially for students’ school well-being (compared to their social inclusion and academic self-concept), low correlations between students’ and teachers’ reports were found. One explanatory factor could be that social inclusion (e.g., contacts with peers) is observed in a better way by the teacher. For the academic self-concept, the high correlation with students’ grades (given by the teacher) might be a possible explanation. Interpreting the trait correlations, the correlations for all three subscales supported the convergent validity of the traits of the PIQ. The findings for discriminant validity indicate high method variance, as the correlations between different traits (same method) were not lower than those between the different rater (same trait). Keeping in mind that the three constructs are not observable variables, this result is not surprising. Teachers have only limited opportunities to observe situations that can be used to infer students’ school well-being, social inclusion, and academic self-concept. In addition, Venetz et al. (2019) found high method-specificity for teacher reports when using students’ reports as a baseline. Moreover, other studies have indicated a low overlap between students’ and teachers’ ratings using other methods. For instance, using socio-metric status, the overlap with teacher attunement is also low (Hoffman et al., 2015). However, the more inclusive a teacher wants to be, the more knowledge he or she needs, which of his or her students are struggling with social inclusion, school-wellbeing, or their academic self-concept to ensure the best socio-emotional development of all students. Therefore for the teacher, it is necessary to listen to the students’ voice and not only decide about support based on his or her own judgment. In this line, further research is needed to better understand the role of the moderating variables. For students (e.g., males or females, students with and without special needs, students with high scores for the outcome variables, or those with a low score), is a high overlap found using the PIQ and do any teacher variables (e.g., years of experience, gender, overall diagnostic competences) predict higher interrater overlap? Moreover, more perspectives could be included, such as the parents’ version of the PIQ, to determine if the rater agreement between students and parents is similar to those of students and teachers.

While the results of the MTMM analysis provided evidence of the convergent and discriminant validity of the traits, they did not support the discriminant validity of the three raters. Only for two methods (students’ and one teacher’s reports), after excluding one, did the teachers’ data from the model confirm the discriminant validity for the raters. Similar results were confirmed by the direct correlation between traits. Evidence of the convergent validity of the traits obtained using different methods indicates latent traits factors that cluster items from the same trait together from different sources (Koch et al., 2015). Furthermore, we found that by having data from only one teacher in addition to students’ data, the possibility of having a latent method factor was reduced. The desired result was to have clear latent traits variables rather than methods latent variables, because the CFA-MTMM uses latent variables to represent both traits and methods (Hintz et al., 2019). Moreover, the current study found a high intraclass correlation for the ratings of both teachers. This could be explained by the fact that reports from two main subject teachers were used. As such, it can be assumed that in the current study, both teachers spent a similar amount of time with all students.

In summary, we propose using both versions of the PIQ (students’ and teachers’ versions). However, including data from a second teacher does not seem to add valuable information. Data from the students and one teacher can explain the data well. The findings demonstrated that the teachers’ version of the PIQ is suitable for use as a screening instrument to assess students’ school well-being, social inclusion, and academic self-concept.

## Data Availability Statement

The datasets generated for this study are available on request to the corresponding author.

## Ethics Statement

The studies involving human participants were reviewed and approved by Ethics Commission of the University of Wuppertal. Written informed consent to participate in this study was provided by the participants’ legal guardian/next of kin.

## Author Contributions

SS and GA designed the study. GA mainly did the calculation part and wrote the results section. SS mainly wrote the other sections of the article. Data collection took place in Germany. All authors contributed to the article and approved the submitted version.

## Conflict of Interest

The authors declare that the research was conducted in the absence of any commercial or financial relationships that could be construed as a potential conflict of interest.

## Footnotes

1 www.piqinfo.ch
